# Supplementary material for: FliO is an evolutionarily conserved yet diversified core component of the bacterial flagellar type III secretion system
Source: Proc Natl Acad Sci U S A. 2025 Aug 21;122(34):e2512476122. doi: 10.1073/pnas.2512476122 (PMC12403147; doi:10.1073/pnas.2512476122)
Supplement: Supplementary file 1 — Appendix 01 (PDF) [file pnas.2512476122.sapp.pdf]

# Supplementary Information

## FliO is an evolutionarily conserved yet diversified core component of the bacterial flagellar type III secretion system

Ekaterina P. Andrianova, Amanda L. Dobbins, Marc Erhardt, David R. Hendrixson, Igor B. Zhulin

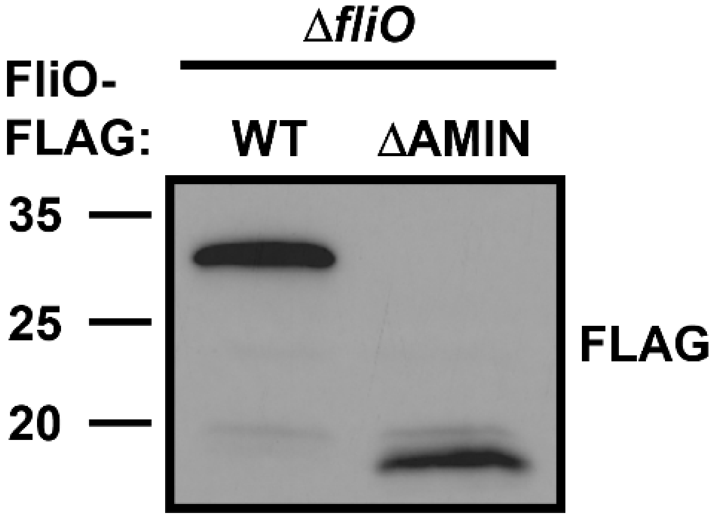


**Supplementary Fig. 1: Immunoblot analysis of immunoprecipitated WT FliO-FLAG and FliO$_{\Delta AMIN}$-FLAG.** *C. jejuni* Δ*fliO* was complemented in the *rrsC* locus with the native *fliNO* promoter expressing *fliO-FLAG* or *fliO*$_{\Delta AMIN}$-*FLAG*. Strains were grown and then diluted to OD$_{600}$ of 0.8. Proteins were immunoprecipitated with a-FLAG resin and, after washing, resuspended in equal volume of 1X SDS-PAGE loading buffer. Equal volumes of samples were examined. a-FLAG antibody was used to detect FliO-FLAG and FliO$_{\Delta AMIN}$-FLAG.

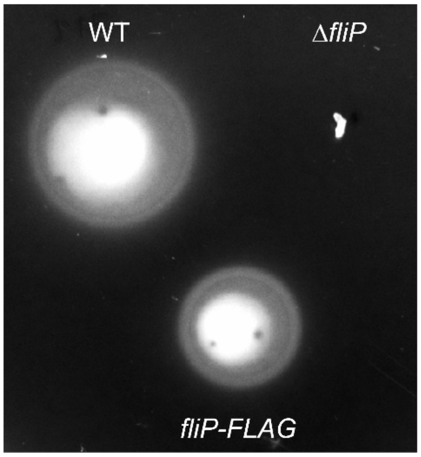


**Supplementary Fig. 2: Characterization of swimming motility of *C. jejuni* strains producing FliP-FLAG.** (A) Representative image of the motility phenotypes of WT *C. jejuni* (top left), Δ*fliP* (top right), and *fliP-FLAG* (bottom). Strains were diluted to an $OD_{600}$ of 0.8 and then stabbed into MH motility agar containing 0.4% agar. Motility was analyzed after 30 h of incubation at 37 °C in microaerobic conditions.

**Supplementary Table 1: Bacterial strains used in this study**

| Strain | Genotype | Source/Reference |
|---|---|---|
| ***E. coli* strains** | | |
| DH5α | *E. coli supE44* Δ*lacU169* (φ80*lacZ*ΔM15) *hsdR17 recA1 endA1 gyrA96 thi-1 relA1* | Invitrogen |
| DH5α / pRK212.1 | DH5α with conjugation transfer element | 1 |
| BL21(DE3) | *E. coli fhuA2 [lon] ompT gal (λ DE3) [dcm]* Δ*hsdS λ DE3 = λ sBamHIo* Δ*EcoRI-B int::(lacI::PlacUV5::T7 gene1) i21* Δ*nin5* | New England Labs |
| | | |
| ***Campylobacter jejuni* strains** | | |
| DRH212 | 81-176 $rpsL^{Sm}$ | 2 |
| DRH706 | 81-176 $rpsL^{Sm}$ *fliP::cat-rpsL* | 3 |
| DRH755 | 81-176 $rpsL^{Sm}$ Δ*fliR* | 3 |
| DRH964 | 81-176 $rpsL^{Sm}$ Δ*flhA* | 3 |
| DRH1065 | 81-176 $rpsL^{Sm}$ Δ*fliP* | 3 |
| DRH8073 | 81-176 $rpsL^{Sm}$ *fliO::cat-rpsL* | This study |
| DRH8113 | 81-176 $rpsL^{Sm}$ Δ*fliO* | This study |
| DAR101 | 81-176 $rpsL^{Sm}$ Δ*fliQ* | 4 |
| SMS508 | 81-176 $rpsL^{Sm}$ *fliQ::cat-rpsL* | 4 |
| SNJ471 | 81-176 $rpsL^{Sm}$ Δ*flhB* | 5 |
| ALD962 | 81-176 $rpsL^{Sm}$ Δ*fliO fliR::kan-rpsL* | This study |
| ALD963 | 81-176 $rpsL^{Sm}$ Δ*fliO fliP::cat-rpsL* | This study |
| ALD965 | 81-176 $rpsL^{Sm}$ Δ*fliO fliQ::cat-rpsL* | This study |
| ALD1050 | 81-176 $rpsL^{Sm}$ Δ*fliO rrsC::kan-*$P_{fliNO}$*-fliO-FLAG* | This study |
| ALD1081 | 81-176 $rpsL^{Sm}$ Δ*fliO rrsC::kan-*$P_{fliNO}$*-*$fliO_{\Delta AMIN}$*-FLAG* | This study |
| ALD1106 | 81-176 $rpsL^{Sm}$ Δ*fliO* $fliP_{P22\text{-}FLAG\text{-}T23}$ | This study |
| ALD1130 | 81-176 $rpsL^{Sm}$ $fliP_{P22\text{-}FLAG\text{-}T23}$ | This study |

**Supplementary Table 2: Plasmids used in this study**

| Plasmid | Genotype | Source/Reference |
|---|---|---|
| pUC19 | $Amp^R$; general cloning vector | New England Biolabs |
| pBR322 | $Amp^R$; general cloning vector | New England Biolabs |
| pILL600 | $Kan^R$; source of *kan* cassette | 6 |
| pDRH265 | pUC19 containing *cat-rpsL* | 3 |
| pDRH643 | pUC19 containing *fliR*::*cat-rpsL* | 3 |
| pDRH645 | pUC19 containing *fliP*::*cat-rpsL* | 3 |
| pDRH2455 | pUC19 containing *fliO* with 0.7 kb upstream and downstream cloned into the BamHI site | This study |
| pDRH2547 | pUC19 with DNA fragment to create an in-frame deletion of codons 2 to 263 of *fliO* cloned into the BamHI site | This study |
| pDRH2559 | pUC19 containing *fliO* with 0.7 kb of upstream and downstream sequence cloned into the BamHI site; contains T410G mutation to create an StuI site in *fliO* | This study |
| pDRH2568 | SmaI-digested *cat-rpsL* cassette cloned into the StuI site of *fliO* in pDRH2559 | This study |
| pDRH7574 | pBR322 with a 2.6 kb DNA fragment containing a portion of the coding sequence of *rrsC* through a portion of *rrlC* was cloned into the EcoRI site | This study |
| pDRH7746 | *kan* cassette from pILL600 cloned into the XbaI site of the *rrsC* locus of pDRH7574 | This study |
| pSMS469 | pUC19 containing *fliQ*::*cat-rpsL* | 4 |
| pALD1047 | *fliNO* promoter from 120 bp up to the *fliN* start codon fused to the start codon of the *fliO* coding sequence with DNA encoding a C-terminal FLAG tag cloned into the XbaI site of pDRH7746 | This study |
| pALD1059 | *fliNO* promoter from 120 bp up to the *fliN* start codon fused to the start codon of the *fliO* coding sequence lacking the predicted AMIN domain (codons 21-118) with | This study |

| | | |
|---|---|---|
| | DNA encoding a C-terminal FLAG tag cloned into the XbaI site of pDRH7746 | |
| pALD1062 | *fliP* locus with 0.7 kb of upstream and downstream sequence with DNA encoding a FLAG tag between codons 22 and 23 to encode FliP$_{P22\text{-}FLAG\text{-}T23}$ cloned into the EcoRI site of pUC19 | This study |

**Supplementary Table 3: Identification by mass spectrometry of fT3SS components isolated from total membrane fractions of WT *C. jejuni* and the Δ*fliO* mutant.**

| Gene ID | Protein Name | WT/Δ*fliO* Abundance* |
|---|---|---|
| CJJ81176_0837 | FliP | 0.79 |
| CJJ81176_0357 | FlhB | 2.26 |
| CJJ81176_0890 | FlhA | 0.94 |
| CJJ81176_0340 | FliF | 1.13 |
| CJJ81176_0341 | FliG | 1.01 |
| CJJ81176_0502 | SecG | 0.99 |

*The ratio of the abundance of each protein in WT *C. jejuni* and *C. jejuni* Δ*fliO* was calculated. FliR and FliQ were not detected by mass spectrometry. SecG is a non-flagellar membrane protein that served as a control for a protein that should be at similar levels in the *C. jejuni* strains.

**REFERENCES**


1. Figurski, D. H. & Helinski, D. R. Replication of an origin-containing derivative of plasmid RK2 dependent on a plasmid function provided in *trans*. *Proc. Natl. Acad. Sci. USA* **76**, 1648–1652 (1979).
2. Hendrixson, D. R., Akerley, B. J. & DiRita, V. J. Transposon mutagenesis of *Campylobacter jejuni* identifies a bipartite energy taxis system required for motility. *Mol. Microbiol.* **40**, 214–224 (2001).
3. Hendrixson, D. R. & DiRita, V. J. Transcription of $\sigma^{54}$-dependent but not $\sigma^{28}$-dependent flagellar genes in *Campylobacter jejuni* is associated with formation of the flagellar secretory apparatus. *Mol. Microbiol.* **50**, 687–702 (2003).
4. Balaban, M. & Hendrixson, D. R. Polar Flagellar Biosynthesis and a Regulator of Flagellar Number Influence Spatial Parameters of Cell Division in *Campylobacter jejuni*. *PLoS Pathog.* **7**, e1002420 (2011).
5. Joslin, S. N. & Hendrixson, D. R. Activation of the *Campylobacter jejuni* FlgSR Two-Component System Is Linked to the Flagellar Export Apparatus. *J. Bacteriol.* **191**, 2656–2667 (2009).
6. Labigne-Roussel, A., Courcoux, P. & Tompkins, L. Gene disruption and replacement as a feasible approach for mutagenesis of Campylobacter jejuni. *J. Bacteriol.* **170**, 1704–1708 (1988).
